# Learning curve in autofluorescence-guided thyroid surgery

**DOI:** 10.3389/fendo.2026.1780244

**Published:** 2026-03-23

**Authors:** Ali Abood, Therese Ovesen, Jacob Bach, Lars Rolighed

**Affiliations:** 1 Department of Otorhinolaryngology, Goedstrup Hospital, Herning, Denmark; 2 Department of Otorhinolaryngology, Hospital South West Jutland, Esbjerg, Denmark; 3 Department of Otorhinolaryngology, Head- and Neck Surgery, Aarhus University Hospital, Aarhus, Denmark

**Keywords:** autofluorescence, hypoparathyroidism, learning curve, parathyroid glands, thyroid surgery

## Abstract

**Background:**

Near-infrared autofluorescence (NIRAF) has been introduced as an adjunct to improve parathyroid gland (PG) identification during thyroid surgery. The extent to which its intraoperative application is influenced by a learning curve remains unclear. This study aimed to evaluate and quantify a potential learning curve in NIRAF-guided thyroid surgery.

**Materials and methods:**

We conducted a retrospective cohort study including all consecutive patients undergoing image-based NIRAF-guided thyroid surgery between 2021 and 2023 at two Danish ENT departments. After each procedure, all surgeons completed a standardized form detailing the manner in which NIRAF was applied. The primary outcome was the minimum number of procedures per surgeon needed to achieve consistent surgical behavior, defined as systematic use of NIRAF or a stable pattern of PG autotransplantation.

**Results:**

A total of 130 patients underwent NIRAF-guided thyroid surgery, performed by two surgeons. Near-infrared autofluorescence was most often utilized on the removed specimen (94.6%), before dissection of the lower thyroid pole (93.1%) and before latero-posterior dissection (88.5%). It was less likely to be utilized before upper thyroid pole dissection (62.3%). For both surgeons, NIRAF application was not systematic in the early phase but became increasingly consistent over time (α = 11,0 (95% CI: 3,5-18,6), p=0,013 and α = 5,6 (95% CI: 0,03-11,2), p=0.049, respectively). Initially, both surgeons demonstrated remarkably high PG autotransplantation rates, which declined over time (α= -3,9 (95% CI: -7,9 - 0,07), p=0,053 and α =-4,8 (95% CI: -8,1 - (-1,6)), p=0.01, respectively). Surgeon no. 1 adopted systematic NIRAF use after a minimum of 30 procedures, while surgeon no. 2 displayed consistent behavior with regard to PG autotransplantation after a minimum of 30 procedures.

**Conclusions:**

This study demonstrates a clear learning curve in NIRAF-guided thyroid surgery. A minimum of 30 procedures per surgeon was required to establish consistent surgical practice, either in terms of systematic NIRAF application or a stable pattern of PG autotransplantation. These findings underscore the importance of structured implementation strategies and targeted training when integrating the NIRAF technology into routine surgical practice.

## 1 Introduction

The application of near-infrared light during thyroid surgery has revealed that parathyroid glands (PGs) appear as distinctly fluorescing subjects, making them easily visible and distinguishable from surrounding tissue (1, 2). This phenomenon is referred to as image-based near-infrared autofluorescence (NIRAF). In other words, the use of near-infrared light during thyroid surgery may substantially facilitate intraoperative PG visualization (3, 4). Consequently, the prerequisites for optimal PG preservation are markedly improved, potentially favoring the mitigation of the most common complication of total thyroidectomy: hypoparathyroidism (5–11).

In recent years, the intraoperative use of NIRAF in thyroid surgery has seen increased traction (12). The technology seems to increase the rate of intraoperative PG identification and reduce the rate of accidental PG removal (13–18). In addition, it allows for early PG identification which improves the prerequisites for preserving PG functionality (19). Ultimately, this is mirrored in lower rates of postoperative hypoparathyroidism, especially in less experienced centers (19).

However, it seems like the reduction in postoperative hypoparathyroidism rates is more evident over time (19). In addition, surgeon behavior also seems to change over time (16, 19). These findings suggest that a learning curve related to the use of NIRAF may be present. Although NIRAF is easy to use, it requires optimal integration in the surgical procedure, as well as correct interpretation of the fluorescence signals. The full impact of NIRAF may therefore not be visible initially as optimal NIRAF utilization first becomes a reality over time. To date, no studies have examined or quantified the role of the learning curve in NIRAF-guided thyroid surgery, thereby underscoring the relevance of such an evaluation.

The aim of this study was to evaluate and quantify a potential learning curve in NIRAF-guided thyroid surgery.

## 2 Materials and methods

A retrospective cohort study was conducted at two low-volume ENT-departments in Denmark (Goedstrup Hospital and Hospital South West Jutland). All consecutive patients who underwent NIRAF-guided thyroid surgery between 2021 and 2023 were identified through the department registries of surgical procedures. Patients were excluded if they underwent surgery without NIRAF guidance or if the surgical procedure was performed by a surgeon who had performed less than 20 NIRAF-guided procedures in total (20, 21). The final cohort thus comprised consecutive patients undergoing NIRAF-guided thyroid surgery performed by surgeons who had each completed more than 20 NIRAF-guided procedures.

All patients had their medical reports reviewed. This included thorough assessments of biochemical profiles (parathyroid hormone (PTH), ionized calcium, thyroid stimulating hormone (TSH), T3, T4) medication histories and pathology reports. All surgeons had postoperatively filled out detailed forms about how NIRAF was used (Appendix I). These forms were assessed as well. Patients who underwent hemithyroidectomy were followed for a minimum of one month postoperatively for complication assessment. Likewise, patients who underwent total thyroidectomy had a follow-up period of up to one year. Hemithyroidectomy was performed by a single thyroid surgeon, while total thyroidectomy normally was performed by two thyroid surgeons (one surgeon removing each thyroid lobe). Individual surgeon trends were evaluated for each surgeon. The number of procedures per surgeon was defined as the total number of procedures in which the surgeon participated (i.e., a total thyroidectomy performed by two surgeons was counted as one procedure for each surgeon). Surgery was performed using the image-based NIRAF device Fluobeam LX (Getinge). Intermittent nerve stimulation was applied in all cases, and magnifying loupes were used upon the surgeon’s request.

The primary outcome was the minimum number of procedures needed per surgeon in order to obtain a consistent surgical behavior. This was assessed by evaluating two parameters: 1) The degree of systematic NIRAF application over time, reflecting how easily NIRAF could be integrated in the surgical procedure, and 2) the pattern of PG autotransplantation over time, as this is reflective of both the integration and the interpretation of NIRAF. Secondary outcomes included operative time, postoperative hypoparathyroidism rates, overall PG identification rates, autotransplantation rates and rates of inadvertent PG excision.

### 2.1 Statistics

Continuous variables were evaluated based on mean values with belonging 95% confidence intervals if a normal distribution was present. Trend assessments were performed using a linear regression model, with α representing the slope of the curve. P-values < 0.05 were considered statistically significant. STATA version 18.0 SE (StataCorp LLC, Texas, USA) was used for data analysis.

## 3 Results

A total of 162 consecutive patients underwent NIRAF-guided surgery performed by eight different surgeons. Thirty-two of these patients underwent surgery by surgeons who performed less than 20 NIRAF-guided procedures (n=6) and were thus excluded. The remaining 130 patients were operated on by two surgeons, each of whom had performed more than 20 NIRAF-guided procedures, and were included in the final analysis. Mean age at the time of surgery was 53.6 years (95% CI: 51.3 – 55.9), with 109 patients (83.8%) being female. Seventy-five patients underwent hemithyroidectomy (57.7%) and the remaining 55 patients underwent total thyroidectomy (42.3%). The overall PG identification rate was 77.0%. Of the identified PGs, 59.3% were found with NIRAF before the naked eye. The rate of inadvertent PG excision was 5.4% and the PG autotransplantation rate was 17.7% (**Table 1**).

**Table 1:** Characteristics of included patients.

Variable	Value
No. of patients	130
Age, mean (95% CI)	53.6 (51.3-55.9)
Sex, female, *n* (%)	109 (83.8)
Body Mass Index, mean (95% CI)	28.3 (27.4-29.3)
Type of surgery	
Hemithyroidectomy, *n* (%)	75 (57.7)
Total thyroidectomy, *n* (%)	55 (42.3)
Indication for surgery	
Symptomatic goiter, *n* (%)	77 (59.2)
Graves’ disease, *n* (%)	26 (20.0)
Diagnostic surgery, *n* (%)	23 (17.7)
Thyrotoxicosis, *n* (%)	4 (3.7)
PG identification and management	
No of PGs identified, *n* (%)	285 (77.0)
No of PGs found with NIRAF before the naked eye, *n* (%)	169 (59.3)
PG autotransplantation, no. of patients, *n* (%)	23 (17.7)
Inadvertent PG excision, no of patients, *n* (%)	7 (5.4)
NIRAF application during surgery:	
Before dissection of upper thyroid pole, *n* (%)	81 (62.3)
Before dissection of lower thyroid pole, *n* (%)	121 (93.1)
Before latero-posterior dissection, *n* (%)	115 (88.5)
On the removed specimen, *n* (%)	123 (94.6)
Systematic application, *n* (%)	66 (50.7)
Complications	
Immediate RLN injury, *n* (%)	3 (2.3)
Permanent RLN injury, *n* (%)	0
Postoperative bleeding, *n* (%)	4 (3.1)
Postoperative infection, *n* (%)	3 (2.3)
Hypothyroidism, *n* (%) ^a^	7 (9.3)
Immediate hypoparathyroidism, *n* (%) ^b,c^	10 (18.2)
Permanent hypoparathyroidism, *n* (%) ^b,d^	4 (7.2)

^a^: Following hemithyroidectomy.

^b^: Following total thyroidectomy.

^c^: Defined as postoperative PTH levels insufficient to maintain normocalcemia without treatment with active vitamin D.

^d^: Defined as PTH levels insufficient to maintain normocalcemia without treatment with active vitamin D one year after surgery.

A detailed assessment of when NIRAF was used during surgery revealed that it was most often utilized on the removed specimen (94.6%), before dissection of the lower thyroid pole (93.1%) and before latero-posterior dissection (88.5%). It was less likely to be utilized before upper thyroid pole dissection (62.3%). Near-infrared autofluorescence was systematically used at all points mentioned above in only 50.7% of cases (**Table 1**).

**Figure 1:**
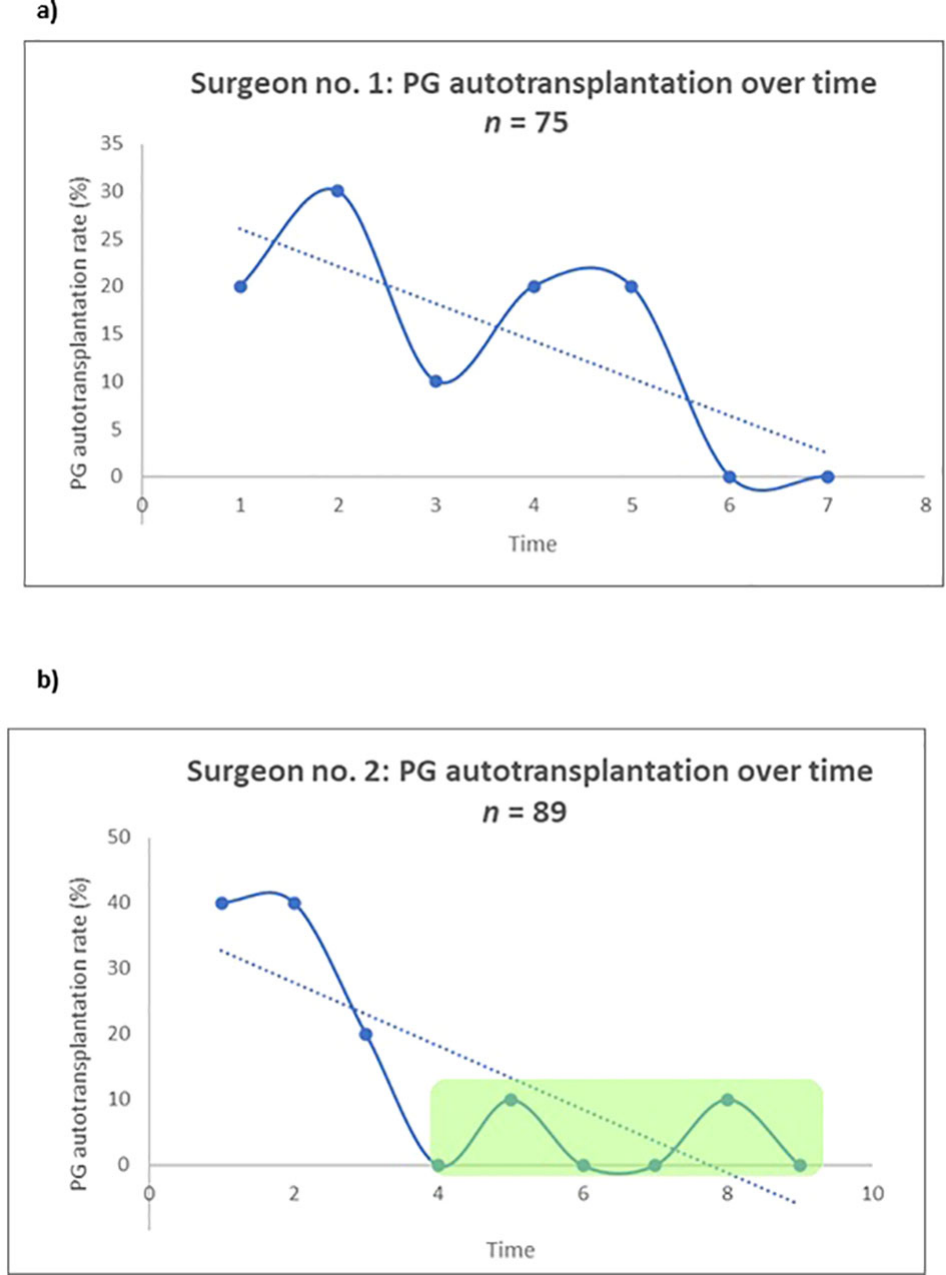
Single-surgeon trends for PG autotransplantation over time for **(a)** surgeon no.1 and **(b)** surgeon no. 2. The surgical procedures were divided into subgroups of 10 consecutive thyroidectomies (both hemithyroidectomy and total thyroidectomy) and PG autotransplantation rates were calculated in each subgroup for **(a)** surgeon no. 1 and **(b)** surgeon no. 2. Note that subgroup no. 7 in **Figure 1a** consisted of 15 patients, and subgroup no. 9 in **Figure 1b** consisted of 9 patients. By linear regression, a fitted line was created in both figures. α **(a)**: -3,9 (95% CI: -7,9 - 0,07), p=0,053. α **(b)**: -4,8 (95% CI: -8,1 - (-1,6)), p=0.01. 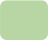: Plateau phase.

**Figure 2:**
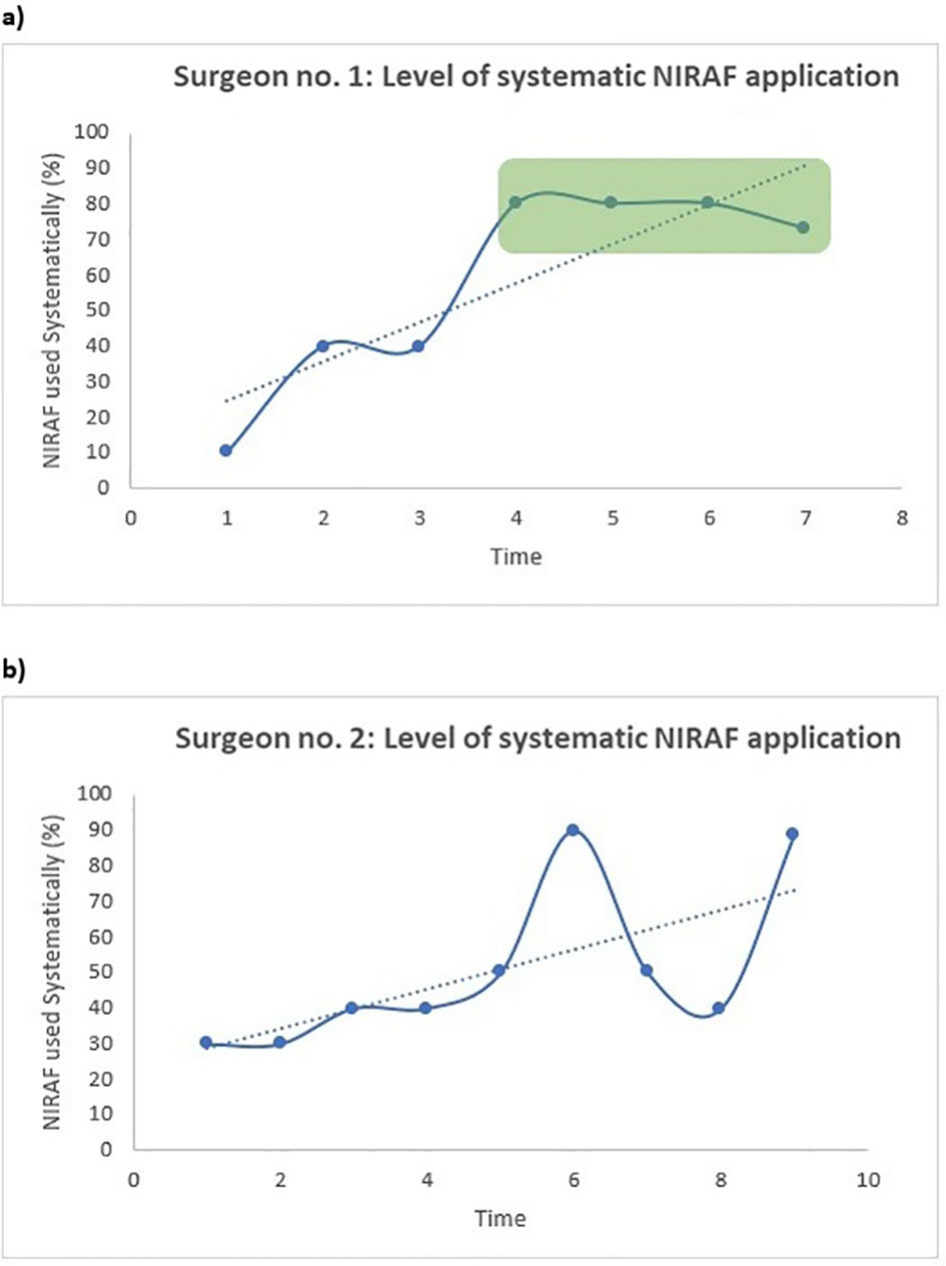
Single-surgeon trends for the level of systematic NIRAF application over time for **(a)** surgeon no.1 and **(b)** surgeon no. 2. The surgical procedures were divided into subgroups of 10 consecutive thyroidectomies (both hemithyroidectomy and total thyroidectomy) and the rates of systematic NIRAF application were calculated for **(a)** surgeon no. 1 and **(b)** surgeon no. 2. Note that subgroup no. 7 in **Figure 2a** consisted of 15 patients, and subgroup no. 9 in **Figure 2b** consisted of 9 patients. By linear regression, a fitted line was created in both figures. α **(a)**: 11,0 (95% CI: 3,5-18,6), p=0,013. α **(b)**: 5,6 (95% CI: 0,03-11,2), p=0.049. 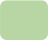: Plateau phase.

**Figures 1** and **2** present the surgical approaches of the two surgeons over time. Specifically, **Figure 1** illustrates the pattern of PG autotransplantation, while **2** depicts the extent of systematic NIRAF application. Surgeon no. 1 participated in 75 thyroid procedures and surgeon no. 2 participated in 89 procedures. Initially, surgeon no. 1 had high autotransplantation rates, reaching up to 30%; over time, these rates declined, approaching 0% at the end (α= -3,9 (95% CI: -7,9 - 0,07), p=0,053). No plateau-phase was confidently reached with regard to PG autotransplantation (**Figure 1a**). Conversely, the rate of systematic NIRAF application was low in the beginning (10%), but increased significantly over time (α = 11,0 (95% CI: 3,5-18,6), p=0,013), reaching a plateau-phase when more than 30 procedures were performed (**Figure 2a**).

Surgeon no. 2 also had high autotransplantation rates in the beginning (40%), followed by a significant decrease over time (α =-4,8 (95% CI: -8,1- (–1,6)), p=0.01). The decrease reached a plateau-phase when more than 30 procedures were performed (**Figure 1b**). Although there also was a significant increase in the rate of systematic NIRAF application over time for surgeon no. 2 (α = 5,6 (95% CI: 0,03-11,2), p=0.049), no plateau phase was reached (**Figure 2b**).

**Figure 3:**
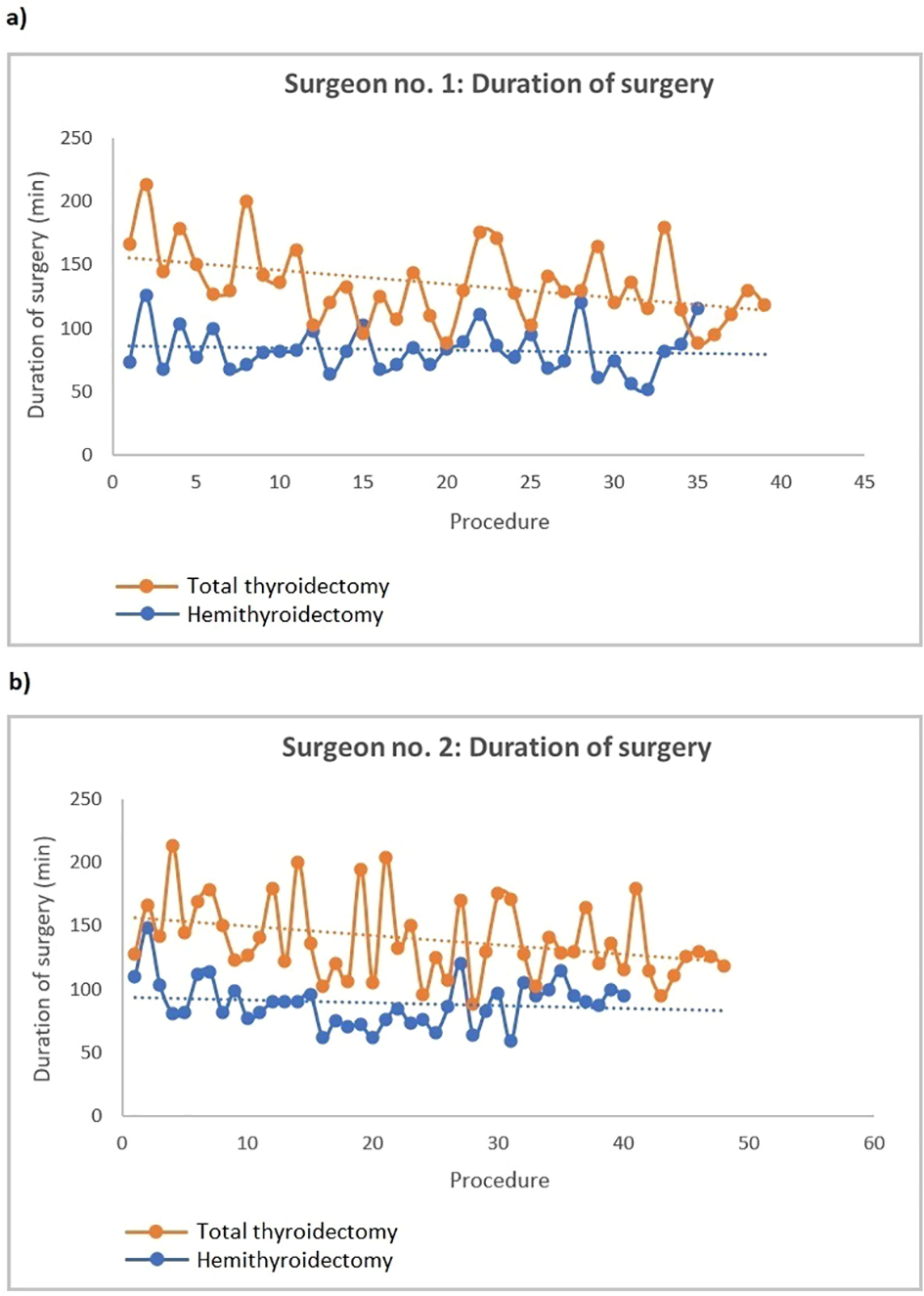
Duration of surgery over time for **(a)** surgeon no.1 and **(b)** surgeon no. 2. By linear regression, a fitted line for both hemithyroidectomy and total thyroidectomy was created for both surgeons. α (surgeon no. 1, hemithyroidectomy): -0.1 (95% CI: -0.3 - 0.2), p=0.606. α (surgeon no. 1, total thyroidectomy): -0.2 (95% CI: -0.3 - (-0.04)), p=0.009. α (surgeon no. 2, hemithyroidectomy): -0.2 (95% CI: -0.7 - 0.3), p=0.400. α (surgeon no. 2, total thyroidectomy): -0.7 (-1.4 - (-0.1)), p=0.021.

**Figure 4:**
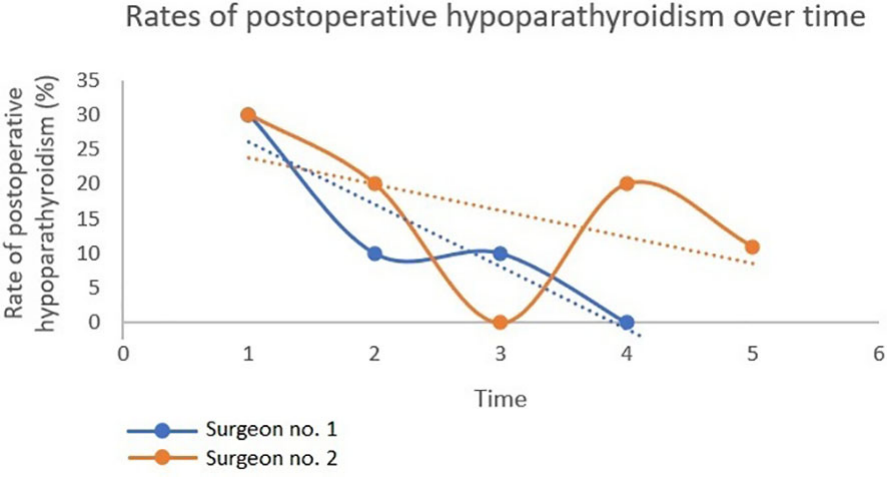
Rates of postoperative hypoparathyroidism over time. Only total thyroidectomies are depicted. For each surgeon, the surgical procedures were divided into subgroups of 10 consecutive total thyroidectomies and the rate of postoperative hypoparathyroidism was calculated in each subgroup. n(surgeon no. 1) = 40 procedures; n(surgeon no. 2)= 49 procedures. Note that subgroup no. 5 (surgeon no. 2) consisted of 9 patients. By linear regression, a fitted line was created for both surgeons. α (surgeon no. 1): -9.0 (95% CI: -20,4 - 2,4, p=0.08). α (surgeon no. 2): -3,8 (95% CI: -14,9 - 7.3, p=0.36).

**Figure 3** depicts the duration of surgery over time. For both surgeons, the duration of surgery in total thyroidectomy significantly declined over time (α = -0.2 (95% CI: -0.3 - (-0.04)), p=0.009) and α = -0.7 (95% CI: -1.4 - (-0.1), p=0.021), respectively). However, no plateau-phase was reached. The duration of surgery in hemithyroidectomy did not change significantly over time for any of the surgeons. The rate of postoperative hypoparathyroidism over time also followed a declining trend for both surgeons, although neither statistical significance nor a plateau-phase was found (**Figure 4**).

## 4 Discussion

In the hitherto first study to quantify the learning curve in autofluorescence-guided thyroid surgery, we found that a consistent surgical behavior was achieved after a minimum of 30 procedures was performed per surgeon. Until that point, surgeon behavior was dynamic. Initially, NIRAF application was unsystematic but became increasingly consistent over time. Conversely, the rate of PG autotransplantation was remarkably high in the early phase but decreased significantly over time. This tendency was also reported in previous studies (16, 19), although no quantification was presented.

The two surgeons evaluated in this study were both experienced surgeons, yet reaching surgical consistency in different ways. After a minimum of 30 procedures, surgeon no. 1 became consistent in terms of how systematically NIRAF was applied. On the other hand, surgeon no. 2 reached consistency with regard to the pattern of PG autotransplantation. While the level of systematic NIRAF application reflects how quickly NIRAF can be integrated in the surgical procedure, the pattern of PG autotransplantation is dependent on both the integration of NIRAF in the surgical procedure and the interpretation of the NIRAF signals. In other words, the two surgeons behaved differently but both surgeons showed a somehow consistent behavior after a minimum of 30 procedures.

It is important to highlight that the findings of our study solely reflect the change in the surgical approach over time. They do not directly define the correct way of using NIRAF, as this would require a link to the change in PG functionality over time. However, the observed decline in the initially high autotransplantation rates may indicate that more PGs were preserved *in situ* over time; especially as the rate of inadvertently excised PGs was very low (5.4%). Arguably, this enhances the prerequisites for preserving PG functionality. The combination of this trend with the observed increase in systematic NIRAF application may suggest that a more systematic use of NIRAF is associated with a higher number of PGs preserved *in situ*.We also evaluated the pattern of postoperative hypoparathyroidism over time for both surgeons. The rates of postoperative hypoparathyroidism tended to decline over time, although without reaching statistical significance. In the same institutions and in the same period (19), hypoparathyroidism rates were initially high in NIRAF-guided surgery, but declined continuously over time, reaching 10% for immediate hypoparathyroidism and 0% for permanent hypoparathyroidism at the end of the study (19). The high rates of hypoparathyroidism in the first year of NIRAF-guided surgery (19) illustrates that the use of NIRAF by itself is not sufficient to prevent hypoparathyroidism. A different and crucial aspect must equally be addressed: Management of PG vasculature. Near-infrared autofluorescence does not provide information about PG vascular supply (22). As PG vessels can be long and possibly creating loops located closely to the thyroid capsule, it is crucial to perform meticulous capsular dissection not only at the site of a PG, but also in the adjacent areas (23). Over time, as more Indocyanine green (ICG)-based studies have emerged, the knowledge about PG vasculature have increased (24–28). This could also have led to a change in surgeon behavior, possibly contributing to the low hypoparathyroidism rates observed at the end of the study (19).

It is worth noticing, that NIRAF was mainly applied on the removed specimen, before dissection of the lower thyroid pole and before latero- posterior dissection. The frequent application of NIRAF on the removed specimen in particular could contribute to the high autotransplantation rates observed initially. Near-infrared autofluorescence was less likely to be used before dissecting the upper thyroid pole, which naturally affects the degree of systematic NIRAF application. A possible explanation for that could be that in some anatomical circumstances it can be difficult to direct the near-infrared light towards the posterior surface of the upper thyroid pole in a satisfactory manner. In that context, application of probe-based NIRAF might be more suitable (29, 30).

The duration of surgery declined significantly over time for both surgeons in total thyroidectomy. However, no plateau-phase was reached. A possible explanation for the lack of a plateau-phase could be that the duration of surgery is highly dependent on the complexity of the case. Complicated cases can occur at any time, resulting in prolonged operative times. It can thus be difficult to demonstrate a consistent plateau-phase using this parameter.

At the end of the study period, the two surgeons stated that they strived to follow the following principles: 1) Systematic and early NIRAF application in order to be able to identify PGs before extensive dissection has been made, thereby increasing the likelihood of *in situ* preservation. 2) Meticulous capsular dissection not only at the site of a PG, but also in the adjacent areas. 3) Treating every PG as if it was the last one. 4) Only performing autotransplantation when a PG is found on the specimen or if it is completely detached in the surgical field (31). These principles were developed throughout the study period as more knowledge was obtained, and have most likely contributed to the low hypoparathyroidism rates observed at the end of the study (19).

The fact that learning curve quantification was based on an analysis of only two surgeons is undoubtfully a limitation of this study. Furthermore, the studies were performed in low-volume institutions where parathyroid surgery is not performed. The external validity of the results must therefore also be limited, as generalizability to a broad spectrum of surgeons cannot be made. However, the results may be reflective of surgeons in similar low-volume, non-parathyroid institutions.

In conclusion, our study demonstrates a clear learning curve in NIRAF-guided thyroid surgery, with a minimum of 30 procedures needed per surgeon to obtain a consistent surgical behavior. NIRAF was not used systematically initially, but the degree of systematic application significantly increased over time. Oppositely, PG autotransplantation rates were remarkably high in the early phase, but showed a pronounced reduction over time. These findings underscore the importance of structured implementation strategies and targeted training when integrating the NIRAF technology into routine surgical practice.

## Data Availability

Dataset can be provided upon request. Requests to access these datasets should be directed to AA, aliahab@outlook.dk.
