# Supplementary material for: Learning curve in autofluorescence-guided thyroid surgery
Source: Front Endocrinol (Lausanne). 2026 Mar 23;17:1780244. doi: 10.3389/fendo.2026.1780244 (PMC13050706; doi:10.3389/fendo.2026.1780244)
Supplement: Supplementary file 1 [file DataSheet1.pdf]

Autofluorescence-guided thyroid surgery

**Procedure:**		
	Extent of surgery:	1. ☐ Hemithyroidectomy<br>2. ☐ Total thyroidectomy
**Autofluorescence:**		
	When was NIRAF applied?	1. ☐ Before dissection of lower pole<br>2. ☐ Before dissection of upper pole<br>3. ☐ Before latero-posterior dissection<br>4. ☐ On the removed specimen
**Parathyroid identification:**		
	Number of parathyroid glands identified:	1 ☐<br>2 ☐<br>3 ☐<br>4 ☐
	Number of parathyroid glands identified with NIRAF before naked-eye identification:	1 ☐<br>2 ☐<br>3 ☐<br>4 ☐
**Autotransplantation:**		
	Number of autotransplanted parathyroid glands:	1 ☐<br>2 ☐<br>3 ☐<br>4 ☐

Weight of specimen: ________g.

Operative time: __________min.

________________________ ________________________

Date **Surgeon(s)**
